# *Sssfh1*, a Gene Encoding a Putative Component of the RSC Chromatin Remodeling Complex, Is Involved in Hyphal Growth, Reactive Oxygen Species Accumulation, and Pathogenicity in *Sclerotinia sclerotiorum*

**DOI:** 10.3389/fmicb.2018.01828

**Published:** 2018-08-07

**Authors:** Ling Liu, Qiaochu Wang, Ying Sun, Yanhua Zhang, Xianghui Zhang, Jinliang Liu, Gang Yu, Hongyu Pan

**Affiliations:** College of Plant Sciences, Jilin University, Changchun, China

**Keywords:** RNA interference, *Sclerotinia sclerotiorum*, SsSFH1, SsMSG5, ROS accumulation, pathogenicity

## Abstract

SFH1 (for Snf5 homolog) protein, comprised in the RSC (Remodels Structure of Chromatin) chromatin remodeling complex, functions as a transcription factor (TF) to specifically regulate gene transcription and chromatin remodeling. As one of the well-conserved TFs in eukaryotic organisms, little is known about the roles of SFH1 protein in the filamentous fungi. In *Sclerotinia sclerotiorum*, one of the notorious plant fungal pathogens, there are nine proteins predicted to contain GATA-box domain according to GATA family TF classification, among which *Sssfh1* (SS1G_01151) encodes a protein including a GATA-box domain and a SNF5 domain. Here, we characterized the roles of *Sssfh1* in the developmental process and fungal pathogenicity by using RNA interference (RNAi)-based gene silencing in *S. sclerotiorum*. RNA-silenced strains with significantly reduced *Sssfh1* RNA levels exhibited slower hyphal growth and decreased reactive oxygen species (ROS) accumulation in hyphae compared to the wild-type (WT) strain. Yeast two-hybrid (Y2H) and bimolecular fluorescence complementation (BiFC) assays demonstrated that SsSFH1 interacts with SsMSG5, a MAPK phosphatase in *S. sclerotiorum*. Furthermore, *Sssfh1*-silenced strains exhibited enhanced tolerance to NaCl and H_2_O_2_. Results of infection assays on soybean and common bean (*Phaseolus vulgaris*) leaves indicated that *Sssfh1* is required for full virulence of *S. sclerotiorum* during infection in the susceptible host plants. Collectively, our results suggest that the TF SsSFH1 is involved in growth, ROS accumulation and virulence in *S. sclerotiorum*.

## Introduction

The Leotiomycetes fungus *Sclerotinia sclerotiorum* (Lib.) de Bary is a necrotrophic plant pathogen that attacks at least 400 described species of plants worldwide. Most of the host plant species are Dicotyledonae, including sunflower, soybean, peanut, lentil and oilseed rape, and only a few of them are Monocotyledonae, such as onion, tulip, and garlic (Boland and Hall, 1994). One of the symbolic features of this notorious fungus is that, at the latter stage of infection, it produces hardened multicellular sclerotium in the form of aggregation of vegetative hyphae (Li et al., 2017). This durable differentiated structure plays a significant role in the survival and persistence of infectious propagules in agriculture fields (Bolton et al., 2006; Yu et al., 2012). Sclerotium can differentiate either into vegetative hyphae or into apothecium, which can produce abundant ascospores that initiate new disease cycles (Bolton et al., 2006). Because of the broad host range, heavy crop loss, and the difficulty of controlling diseases caused by *S. sclerotiorum*, sustained research efforts have focused on this pathogen (Xiao et al., 2014).

*S. sclerotiorum*, as a broad host range necrotrophic fungus, has evolved sophisticated and comprehensive strategies to infect host plants (El Oirdi et al., 2011; Kabbage et al., 2015). Initially, *S. sclerotiorum* directly penetrates the healthy plant cuticle with modified hyphae, which ranging from single-celled to multicellular compound appressoria to infection cushions (Li et al., 2012). The infection cushions impose mechanical pressure to penetrate host plant cells, eventually leading to plant disease (Uloth et al., 2016). During the establishment of pathogen-host interactions, some specific and non-specific defense responses in plants against invading pathogens are triggered; leading to reactive oxygen species (ROS) burst, plant cell death and some other responses. Both plants and pathogens could produce ROS around the infection site (Egan et al., 2007). It was reported that ROS could accumulate in the developing and mature appressoria or fungal penetration structures, and the appropriate regulation of ROS production plays important roles in developmental growth and virulence of fungal pathogens (Lambeth, 2004; Egan et al., 2007; Ryder et al., 2013). Necrotrophic pathogens thrive on the dead host tissues that they kill before or during colonization (Horbach et al., 2011), so the host cell death might be beneficial rather than detrimental for necrotrophic pathogens (Lyu et al., 2016).

SFH1 (for Snf5 homolog) is one of the RSC (Remodels Structure of Chromatin) complex protein, which is highly similar to the SWI/SNF (SWItch/Sucrose Non-Fermentable) subunits SNF5 (Sucrose non-fermenting) protein (Hsu et al., 2003). RSC is an ATP-dependent remodeling complex and is essential for viability and cell cycle progression (Hsu et al., 2003; Campsteijn et al., 2007; Monahan et al., 2008). Studies in yeast implicated that RSC plays roles in the cellular response to stress and RSC executes functions in G1 phase in addition to its essential roles in G2/M phase (Damelin et al., 2002; Ng et al., 2002; Campsteijn et al., 2007). Furthermore, in yeast, mutation in *sfh1* arrests the cell cycle (Cao et al., 1997), and the centromeric chromatin structure is altered in this mutant (Hsu et al., 2003). Mutant forms of the human ortholog of *sfh1*, the tumor suppressor INI1/hSNF5, can induce the appearance of tetraploid cells (Zhang et al., 2002; Vries et al., 2005). Though the roles of SFH1 in other eukaryotic organisms are well defined, the role of SFH1 protein in filamentous fungus is rarely reported.

In *S. sclerotiorum*, the *Sssfh1*, orthologous to the *S. cerevisiae sfh1*, is predicted to encode a GATA-box and a SNF5 domain-containing transcription factor (TF). In this study, we characterize the biological and molecular functions of *Sssfh1* using RNA interference (RNAi) strategy. Similar to its counterpart, SFH1 in yeast, this predicted SsSFH1 protein in *S. sclerotiorum* exhibits specific functions in proper (hyphal) cell growth. We also demonstrate this protein takes parts in ROS accumulation and fungal pathogenicity.

## Results

### SsSFH1 Is a Component of the RSC Protein Complex

**FIGURE 1:**
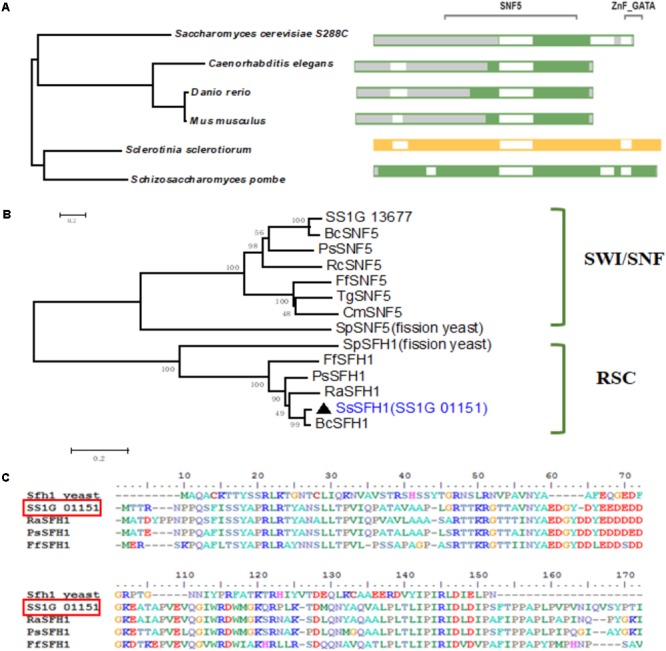
Sequence and phylogenetic analysis of SsSFH1. **(A)** SmartBLAST with NCBI: The query sequence SsSFH1 was highlighted in yellow; the amino acids, which are not aligned to the query sequence are shaded in gray. The gap is in white; landmark matches are shaded in green. Sequences are from *S. sclerotiorum, Danio rerio, Mus musculus, Schizosaccharomyces pombe, Saccharomyces cerevisiae* S288c and *Caenorhabditis elegans*. **(B)** Phylogenetic analysis of the conserved domain from different species. The selected proteins are: *S. sclerotiorum*, SsSFH1 (XP_001596958.1) and SS1G_13677 (XP_001585438.1); *Fusarium fujikuroi*, FfSFH1 (KLP21684.1) and FfSNF5 (KLP22090); *Botrytis cinerea*, BC1G_12110 (XP_001549133.1) and BcSNF5 (XP_001546059.1); *Phialocephala subalpina*, PsSFH1 (CZR60918.1) and PsSNF5 (CZR66927.1); *Rhynchosporium agropyri*, RaSFH1 (CZT00122.1); *Rhynchosporium commune*, RcSNF5 (CZT07508.1); *Cordyceps militaris*, CmSNF5 (ATY60809.1); *Trichoderma guizhouense*, TgSNF5 (OPB42243.1); and *Schizosaccharomyces pombe*, SpSFH1 (NP_588001.1) and SpSNF5 (NP_592979.1). The proteins are grouped into fungal SWI/SNF and RSC. Phylogenies were speculated using MEGA (version 7.05) program with the Neighbor-Joining method (1000 bootstrap replicates) to create an unrooted phylogenetic tree. **(C)** Sequence alignment of SNF5 domain from representative SFH1 proteins: *S. sclerotiorum*, SsSFH1 (XP_001596958.1), *Fusarium fujikuroi*, FfSFH1 (KLP21684.1), *Phialocephala subalpina*, PsSFH1 (CZR60918.1), *Rhynchosporium agropyri*, RaSFH1 (CZT00122.1), and *Schizosaccharomyces pombe*, Sfh1 (NP_588001.1). The sequence alignment was performed with BioEdit program.

GATA family TFs contain two zinc finger motifs and bind specifically to the DNA sequence (A/T) GATA (A/G) (Chi et al., 2013). According to GATA family TF classification, there are nine proteins predicted to contain GATA-box domain in *S. sclerotiorum* (Amselem et al., 2011). Besides SsNSD1, other GATA TFs are not well-studied (Li et al., 2017). We isolated a gene (SS1G_01151) encoding a TF containing a GATA-box and a SNF5 domain (**Supplementary Figure S1A**). The SmartBLAST server, which can be used to quickly find the closest homologs to the protein, was used in our study. In our analysis, this GATA-box and SNF5 domain-containing protein in *S. sclerotiorum* is orthologous to the SFH1 proteins from *Danio rerio, Mus musculus, Caenorhabditis elegans, Saccharomyces cerevisiae* S228C, and *Schizosaccharomyces pombe* (**Figure 1A**). We also performed sequence alignment and phylogenetic analysis using GATA-box and SNF5 domain-containing proteins from different fungi species, such as *Fusarium fujikuroi, Botrytis cinerea, Phialocephala subalpina, Rhynchosporium agropyri, Rhynchosporium commune, Cordyceps militaris, Trichoderma guizhouense*, and *S. pombe*, and the results showed that this SNF5 domain-containing TF (SS1G_01151) belongs to the RSC proteins complexes clade (**Figures 1B,C**). Based on the results from SmartBLAST search and phylogenetic analysis, this putative SNF5 domain-containing TF in *S. sclerotiorum* was grouped to the RSC subfamily and was named as SsSFH1.

### Molecular Characterization of *Sssfh1*-Silenced Strains

**FIGURE 2:**
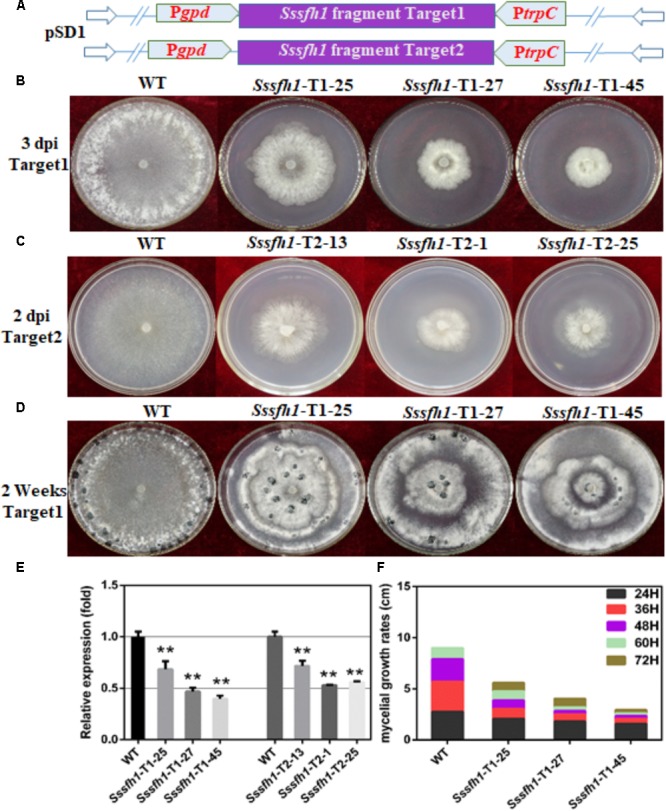
Biological characterization of the *Sssfh1-*silenced transformants. **(A)** Diagrams of constructs used to silence *Sssfh1* in *S. sclerotiorum.* **(B,C)** The hyphal growth of WT, *Sssfh1*-Target1-silenced strains and *Sssfh1*-Target2-silenced strains. Selected Target1 transformants and WT strain were inoculated on PDA plates and the pictures were taken 3 days post-inoculation (dpi) at 25°C, and the pictures of Target2 transformants were taken 2 dpi. **(D)** Sclerotial morphology of the WT and RNAi strains. Transformants and WT strain were cultured on PDA plates and grown at 25°C. Pictures were taken 14 dpi. **(E)** Relative expression level of *Sssfh1* in different RNAi strains. Transformants and WT strain were inoculated on PDA media for two days and the hyphae were collected for RNA preparation. A value of one was assigned to the relative abundance of *Sssfh1* in the WT strain. The gene expression levels of *Sssfh1* in the silenced transformants and the WT strain were normalized to that of the mean of tubulin, actin, and histone transcripts from each strain. Data = means ± SD, one-way ANOVA, ^∗∗^indicates significance at *p* < 0.01. **(F)** Hyphal growth rates of the RNAi strains and the WT strain cultured on PDA plates. The first measurement began at 24 hpi, and the interval was set as every 12 h. The measurement was completed at 72 hpi. Three independent biological replications were performed.

To determine the function of *Sssfh1*, RNAi strategy was used to knock down its transcriptional expression. *S. sclerotiorum* is a typical multi-nucleated fungus, which makes it difficult to generate homozygous and pure knockout strains, and the RNAi technique could be a good complementary approach to investigate gene function in this fungus (Lyu et al., 2016). We performed BLASTN search and found low similarities between *Sssfh1* and others genes in *S. sclerotiorum* genome (**Supplementary Figure S1B**). To increase our confidence on the RNAi efficiency and to minimize the stability issues caused by this technique, we designed two sets of RNAi constructs (no overlapping), targeting two different *Sssfh1* coding regions, based on the pSilent-Dual1 system (Nguyen et al., 2008), resulting in pSD-*Sssfh1-*Target1 and pSD-*Sssfh1-*Target2 constructs (**Figure 2A**). The resulting constructs were subsequently introduced into *S. sclerotiorum* through polyethylene glycol (PEG)-mediated protoplast transformation (Rollins, 2003). The antibiotics resistant transformants were subjected to the examination of the presence of antibiotics marker gene by PCR (**Supplementary Figure S2**). *Sssfh1* silencing strains generated from both constructs described above showing similar hyphal growth phenotypes ensure us that the RNAi strategy we exploited caused little off-target effect in our experiment (**Figures 2B,C** and **Supplementary Figure S2**). We analyzed the transcript accumulations of *Sssfh1* in different strains by qRT-PCR and the results suggested that both constructs could be used to efficiently reduce the accumulation of *Sssfh1* transcripts in the selected transformants (**Figure 2E**).

### *Sssfh1* Is Required for *S. sclerotiorum* Hyphal Growth, Development and Sclerotial Development

To determine the role of *Sssfh1* in *S. sclerotiorum* hyphal growth, we compared the aerial hyphal growth of the WT and RNAi strains. The results showed that RNAi strains showed slower hyphal growth compared to the WT strain, which is likely correlated with their *Sssfh1* expression levels (**Figures 2B,C,E**). Due to the RNAi strains from two different targets showed similar gene silencing efficiency and the impaired hyphal growth phenotype, the RNAi transformants from Target1 was used for the subsequential experiments. Colony diameters of the WT and RNAi strains on Potato Dextrose Agar (PDA) medium were measured to further compare the growth of each strain and the results showed that the hyphal growth was highly reduced in *Sssfh1*-silenced strains (**Figure 2F**). These results suggest that *Sssfh1* plays an important role in aerial hyphal growth. Apart from the hyphal growth, under the same conditions, the *Sssfh1-*silenced strains showed affected distribution of the sclerotia at the center of the plate and produced less numbers of sclerotia than did the WT strain (**Figure 2D** and **Supplementary Figure S3**).

**FIGURE 3:**
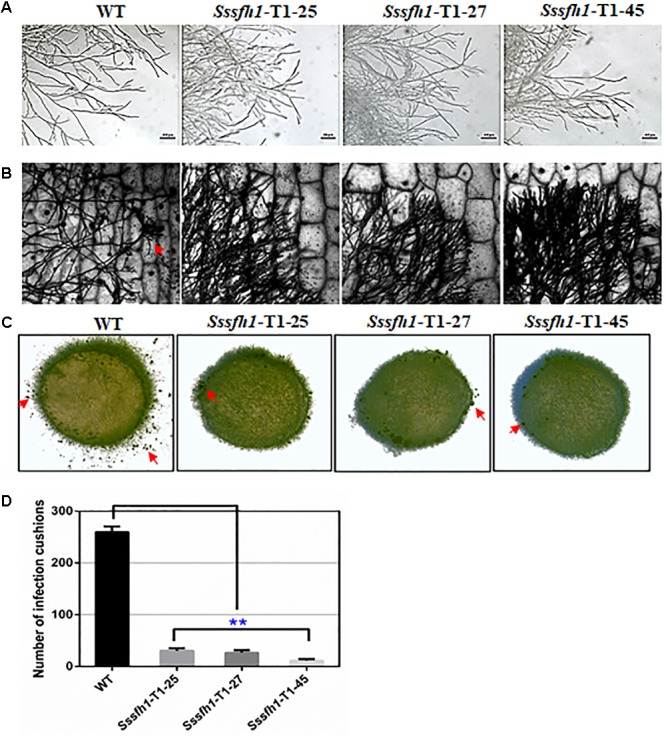
Hyphal and appressorium development in *S. sclerotiorum*. **(A,B)** Morphological comparison of hyphal tips from the *Sssfh1*-silenced transformants and the WT strain. Mycelial plugs of WT and *Sssfh1*-silenced strains were inoculated on CM plates **(A)** or on onion epidermis **(B)** for 24 h before observation. **(C)** Infection cushion production of WT and *Sssfh1*-silenced strains. Pigmented infection cushions (the red arrow) were observed on the glass slide at 24 hpi using mycelia plugs (5 mm in diameter). The photographs were taken with a Nikon SMZ1500 light microscope. **(D)** Data analysis in **(C)**. Data = means ± SD, one-way ANOVA, ^∗∗^indicates significance at *p* < 0.01. Three independent replications were performed.

The formation of hyphal branches from WT and RNAi strains were observed under optical microscope and we found that RNAi strains showed abnormal hyphal branches on both PDA medium and onion epidermis (**Figures 3A,B**). Compared with the WT strain, the hyphal tip from RNAi strains are denser and are more likely to adhere to each other (**Figures 3A,B**). In order to investigate whether *Sssfh1* influences multicellular compound appressoria (infection cushions) development in *S. sclerotiorum*, we examined infection cushion development in the WT and RNAi strains. In this assay, the WT strain rapidly differentiated infection cushion structures from vegetative hyphae upon contact with microslide (**Figure 3C**), while fewer similar differentiated structures were observed in the RNAi strains (**Figures 3C,D**).

### *Sssfh1*-Silenced Transformants Exhibit Impaired Pathogenicity

**FIGURE 4:**
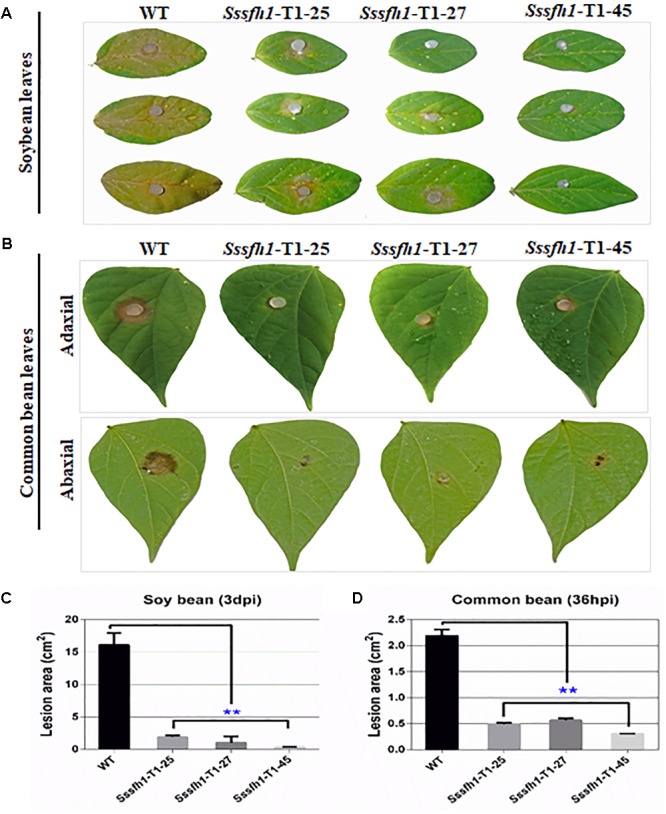
*Sssfh1* is required for the full virulence of *S. sclerotiorum*. **(A)** The *Sssfh1-*silenced strains show the impaired capacity to invade soybean leaves. Mycelial plugs of the indicated strains were inoculated on soybean leaves and disease symptoms were observed and photographed at 3 dpi. This experiment was repeated three times. **(B)** *Sssfh1-*silenced strains caused reduced disease lesions on common bean leaves. Symptoms were photographically documented at 36 hpi from both adaxial (upper panel) and abaxial leaf epidermis (lower panel). **(C,D)** Quantitative analysis of the lesion sizes in **(A,B)**. Data represent means ± SD from at least three independent experiments (one-way ANOVA, ^∗∗^indicates significance at *p* < 0.01).

We show that reducing the expression of *Sssfh1* could negatively affect the hyphal growth and infection cushions formation (**Figures 2, 3**). To unravel whether *Sssfh1* is also responsible for the full virulence of this notorious plant pathogen, the agar plugs from either WT control or RNAi strains were inoculated on the detached soybean leaves. Compared to the fully pathogenic WT strain, the RNAi strains produced smaller disease lesions, suggesting that the infection capacity was highly impaired in the *Sssfh1*-silenced strains (**Figures 4A,C**). Similar phenotypes were observed when we performed the same inoculation assay using common bean, another host plant of this fungal pathogen (**Figures 4B,D**). In parallel, tomato leaves were also inoculated with these strains and smaller necrotic lesions were observed, which were produced by the RNAi strains compared to WT strain (**Supplementary Figure S4A**). In the pathogen inoculation assay, we found a good correlation between disease lesion sizes and gene expression levels, which varied in different transformants (**Figures 2E, 4** and **Supplementary Figure S4**). To determine whether the infection cushion deficiency of RNAi strains contributed to the impaired pathogenicity, on wounded host tissues, infection assays were performed. Similar disease phenotypes were observed that the RNAi strains caused smaller necrotic lesions on wounded leaf tissue compared to the WT strain (**Supplementary Figure S4B**). Together with the less infection cushion formation in RNAi strains, these results suggested that the normal transcript accumulation of *Sssfh1* is required for normal infection cushion formation and full virulence in *S. sclerotiorum*. To monitor the characteristics of the lesions in WT and RNAi strains-infected plant tissues, pathogen-inoculated common bean leaves were sampled 24 h after pathogen inoculation (**Supplementary Figure S5A**) and were decolorized prior to microscopy. The microscopy observation results of those infected areas showed that *Sssfh1*-silenced strains rarely produced massive infection sites. However, at the same time point the WT strain infectious hyphae could penetrate the host plant successfully, and inter- and intracellular infectious hyphae have formed within host cells and gave rise to necrosis within the infection scale (**Supplementary Figures S5B,C**).

### SsSFH1 Interacts With SsMSG5

**FIGURE 5:**
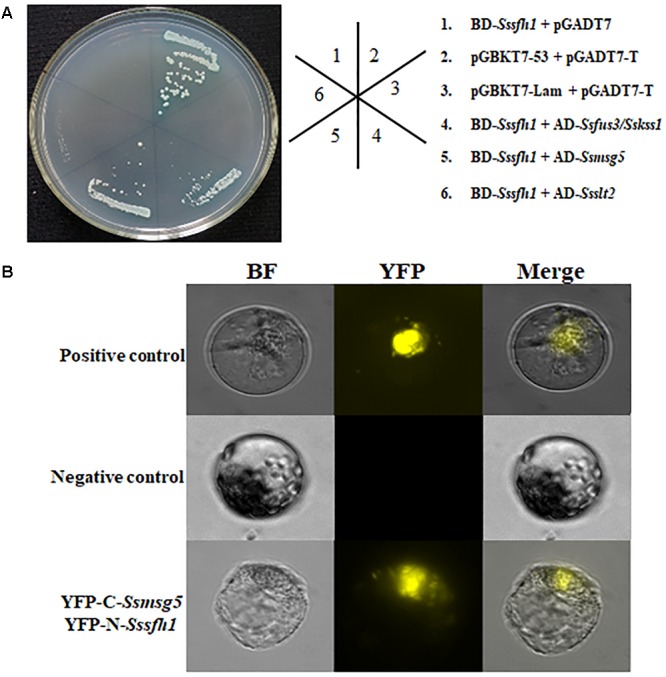
SsSFH1 interacts with SsMSG5. **(A)** Yeast two-hybrid assay reveals that SsSFH1 interacts with SsMSG5 in AH109 yeast cells. *Sssfh1* was inserted into pGBKT7 and the *Ssmsg5, Ssfus3/Sskss1*, and others were inserted into pGADT7. Co-transformed AH109 cells were streaked on the SD/-Leu-Trp or SD/-Leu-Trp-His-Ade with X-α-Gal. pGBKT7-Lam and pGADT7 were used as negative control, while pGBKT7-53 and pGADT7-T were used as positive control. pGBKT7-*Sssfh1* and pGADT7 were used for self-activation detection. **(B)** The interaction between SsSFH1 and SsMSG5 was confirmed by BiFC assay in *Arabidopsis* protoplast. The well-known interaction proteins AtCRY2 and AtCIB1 were used as positive control, while the empty cYFP and SsSFH1-nYFP were used as negative control. One representative data is shown from at least three independent experiments.

To further understand the molecular function of *Sssfh1* affecting the development of *S. sclerotiorum*, the yeast two-hybrid (Y2H) system was used to identify SsSFH1-interacting proteins. We used *Sssfh1* in pGBKT7 vector as bait to identify interacting proteins against the *S. sclerotiorum* cDNA library. Six putative positive clones were obtained after initial screening. To confirm the interactions, each putative interactor in the prey plasmid was co-transformed with either pGBKT7*-Sssfh1* bait plasmid or pGBKT7 plasmid (control) into AH109, and the transformed yeast cell were grown on SD/-Ade/-His/-Leu/-Trp/X-α-Gal plates to increase the confidence of the co-transformation. In our screening, only two SsSFH1-interating proteins, SsMSG5 (SS1G_06950) and SsFUS3/SsKSS1 (SS1G_11866), were obtained as indicated by blue pigment production under these selection conditions (**Figure 5A**), and the SsSLT2 (SS1G_05445) was found to have only a weak interaction with SsSFH1 (data not shown). We then tried to confirm the interactions between SsSFH1 and SsMSG5 or SsFUS3/SsKSS1 using the bimolecular fluorescence complementation (BiFC) technique in *Arabidopsis* protoplast system (Wei et al., 2016). In BiFC assay, SsSFH1-nYFP interacted with SsMSG5-cYFP, resulting in yellow fluorescence in protoplast under microscopy (**Figure 5B**), but SsSFH1-nYFP and SsFUS3/SsKSS1-cYFP combination did not produce fluorescence in protoplast (**Figure 5B**). Here we conclude that SsSFH1 interacts with SsMSG5. We then examine the *Ssmsg5* gene transcriptional level in *Sssfh1*-silenced strains by qRT-PCR and the results showed that the transcription of *Ssmsg5* was reduced in RNAi strains (**Supplementary Figure S6**).

### *Sssfh1* Is Involved in Osmotic and Oxidative Stress Responses

**FIGURE 6:**
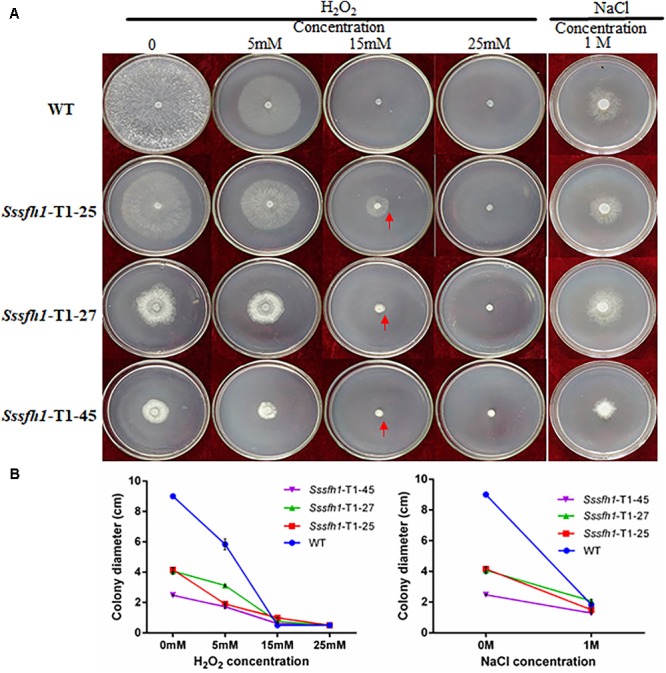
*Sssfh1*-silenced strains show increased tolerance to osmotic and oxidative stresses. **(A)** Representative pictures showing that RNAi strains are more tolerant to osmotic and oxidative stresses. Both WT and RNAi strains were inoculated on PDA media that was supplemented with sequential concentrations of oxidative and osmotic reagents, in which H_2_O_2_ (5, 15, and 25 mM) was used to generate oxidative stress and 1 M NaCl was used to mimic osmotic stress environment. Photographs were taken after incubation at 25°C for 3 days. **(B)** The colony diameters of different strains in A. Each data point represents the means ± SD from three biological repeats (*n* = 3).

SsSFH1 interacts with SsMSG5 (**Figure 5**) and MSG5 in yeast was reported to interact with and dephosphorylate the activated form of SLT2, which is related to oxidative stress (Zhang et al., 2017). We then tried to examine whether the alteration of *Sssfh1* transcripts could affect hyphal cell response to osmotic and oxidative stress by challenging both WT and RNAi strains with different osmotic stress (NaCl) and oxidative stress (H_2_O_2_) reagents. In the presence of 1 M NaCl in the PDA media, the relative inhibition rates of hyphae growth of *Sssfh1* RNAi strains were significantly lower than the WT strain. *Sssfh1* RNAi strains less sensitive to the NaCl compared with the WT strain (**Figures 6A,B** and **Supplementary Figure S7**). When the PDA media was amended with varying concentrations of H_2_O_2_ from 5 to 25 mM, intriguingly, our results showed that the less growth inhibition of the RNAi strains than that of the WT strain, indicating that silencing *Sssfh1* gene made *S. sclerotiorum* more tolerant to higher concentrations of exogenous H_2_O_2_ (**Figures 6A,B**). This result indicated that SsSFH1 is likely to be a negative regulator in *S. sclerotiorum* in response to osmotic stress and oxidative stress.

### Silencing *Sssfh1* Influences ROS Accumulation in *S. sclerotiorum*

**FIGURE 7:**
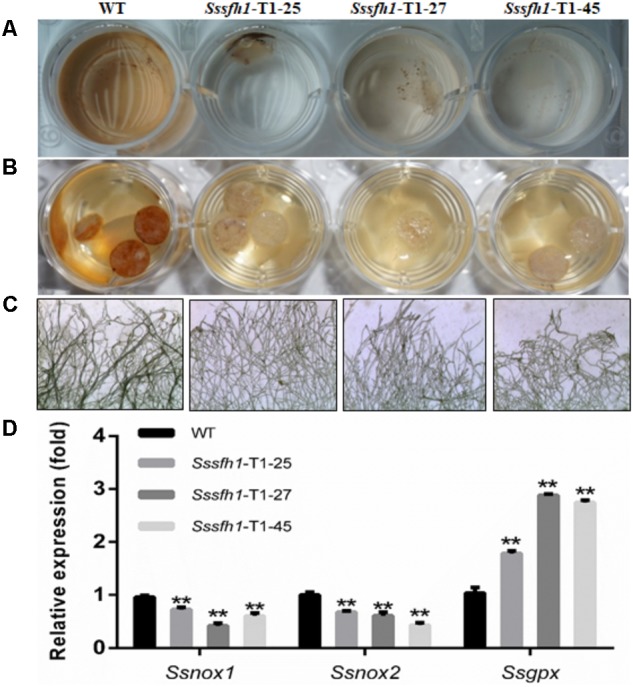
Silencing *Sssfh1* alters ROS accumulation in *S. sclerotiorum*. **(A)** DAB staining of detecting ROS accumulation (H_2_O_2_) in different strains. The mycelium from different strains was treated with DAB staining to determine the generation of H_2_O_2_. DAB staining was treated for 2 h and the images were then taken. **(B)** The mycelial plugs from different strains were taken at 36 h post PDA media inoculation and were stained with DAB staining. DAB staining was treated for 2 h and the images were taken. The different intensities of brown coloration represent different levels of ROS accumulation. **(C)** Detailed marginal mycelia morphology after NBT staining. The marginal mycelia were from the same culture as in B. The images indicate that the *Sssfh1*-silenced strains accumulate less ROS (O_2_^-^) as revealed by less stained mycelia after NBT staining for 20 min. The effect of NBT staining of different mycelium was observed with a light microscope. The different intensities of blue precipitate represent different levels of ROS (O_2_^-^) accumulation. **(D)** Expression levels of ROS accumulation-related genes. The expression of NADPH oxidase (NOX) genes *Ssnox1, Ssnox2*, and glutathione peroxidase (GPX) gene *Ssgpx* were investigated by qRT-PCR assay in hyphae tissue in RNAi and WT strains. Each data point represents the means ± SD from three biological repeats (*n* = 3) (one-way ANOVA, ^∗∗^indicates significance at *p* < 0.01).

Osmotic stress and oxidative stress are often associated with ROS production (Segmuller et al., 2007). Also, as a necrotrophic pathogen, *S. sclerotiorum* triggers massive ROS burst and hypersensitive cell death in the host and the appropriate ROS accumulation is of importance in *S. sclerotiorum* pathogenicity (Kim et al., 2011). To further detect ROS production by different strains, the same amount of fresh mycelia or the mycelia plugs of WT and RNAi strains grown on PDA were stained with 3,3*N*-Diaminobenzidine Tetrahydrochloride (DAB) solution. The lighter brown coloration of the mycelia from RNAi strains compared to WT strain suggested a decreased accumulation of H_2_O_2_ among RNAi strains in normal growth condition (**Figures 7A,B**). Similarly, the formation of O_2_^-^ was monitored by treating mycelia from WT and RNAi strains with nitro blue tetrazolium (NBT). The extensive precipitation of blue formazan was observed in the WT hyphae, while much less blue formazan precipitation was observed in RNAi strains, which demonstrated that reducing the *Sssfh1* transcript accumulation altered the net accumulation of O_2_^-^ (**Figure 7C**). Regulation of ROS synthesis by NADPH-dependent oxidase complex (NOX) is necessary in fungi (Lara-Ortiz et al., 2003; Kim et al., 2011), and the enzymatic activity of GPX is responsible for ROS scavenging, which converts H_2_O_2_ to H_2_O (Heller and Tudzynski, 2011). To determine whether the expression patterns of *Ssnox1* (SS1G_05661), *Ssnox2* (SS1G_11172), and *Ssgpx* (SS1G_00741) were altered in RNAi strains, we profiled the expression of these three genes with qRT-PCR assay. Our results demonstrated that, in RNAi strains, *Ssnox1* and *Ssnox2* showed a lower transcriptional accumulation than did in the WT strain, conversely, the higher expression of *Ssgpx* was detected in those RNAi strains (**Figure 7D**). Collectively, our results suggest that reducing the expression of *Sssfh1* might negatively affect the ROS accumulation in *S. sclerotiorum* hyphae in a possible manner of reducing the expression of ROS production-responsible genes and increasing the expression of ROS scavengers.

## Discussion

In *S. cerevisiae, sfh1* was identified as a component of RSC complex that is required for cell cycle progression (Cao et al., 1997). *Sfh1* knockout strains in *S. cerevisiae* exhibit abnormal phenotypes, such as cell cycle arrest and altered mitotic growth (Bonazzi et al., 2005), while disruption of *sfh1* in fission yeast makes the cells lethal at both 22 and 33°C (Monahan et al., 2008). It was also reported that the core centromeric chromatin and flanking chromatin structures were significantly altered in *sfh1* mutants in *S. cerevisiae* (Hsu et al., 2003). In this study, our sequence analysis of SsSFH1 reveals that, besides the SNF5 domain, SsSFH1 contains a highly conserved GATA-box (**Figure 1** and **Supplementary Figure S1**). Many proteins with GATA-box have a wide range of functions, from terminal differentiation in vertebrates (Tsang et al., 1997; Perlman et al., 1998; Charron et al., 1999) to nitrogen metabolism, siderophore biosynthesis, photoinduction, and mating type switch in fungi (Scazzocchio, 2000). According to the roles of SFH1 orthologs, we hypothesized that *Sssfh1* may play roles in fungal growth and development in *S. sclerotiorum.* We tried to knock out *Sssfh1* by homologous recombination-based gene replacement as we did in our previous studies (Wang et al., 2016; Li et al., 2017), and no antibiotic resistance positive transformant was obtained in our standard gene knockout procedure. Considering the roles of *sfh1* in yeast cells, which is involved in the process of cell cycle, chromatin remodeling (Cao et al., 1997), *Sssfh1* knockout might be lethal (Monahan et al., 2008) in *S. sclerotiorum*. We used RNAi as an alternative strategy to investigate the function of *Sssfh1* in *S. sclerotiorum*, instead of total removal of its transcript, in a way of reducing the transcript accumulation. Compared with the normal growth in WT strain, the *Sssfh1*-silenced strains exhibited altered growth and developmental phenotypes, such as slower hyphal growth and development, as well as sclerotia development (**Figures 2, 3**). As an important agricultural pathogen, *S. sclerotiorum* infects hundreds of species of plant, including lots of crop species (Guyon et al., 2014). We inoculated the RNAi and WT strains on different plant species and the results suggested that reducing the expression of *Sssfh1* leads to the impairment of pathogenicity of this notorious pathogen in the conditions of both healthy and wounded detached plant leaves (**Figure 4** and **Supplementary Figure S5**). These results indicate that *Sssfh1*, besides its important role in growth and development, is also involved in the full virulence of *S. sclerotiorum*.

In our yeast two-hybrid screening, MSG5 (Multicopy Suppressor of the G protein alpha subunit GPA1), removal of which in yeast genome causes slower growth rate and a nearly complete cell cycle arrest (Andersson et al., 2004), was identified as an interactor of SsSFH1. This interaction was further confirmed by Y2H and BiFC assays (**Figure 5**). *Ssmsg5* encodes a phosphatase that influences the mitogen-activated protein kinase KSS1-dependent filamentation pathway in *S. cerevisiae* and plays roles in cell cycle (Flandez et al., 2004; Palacios et al., 2011). In yeast cells, SFH1 is phosphorylated and the phosphorylation of this protein is regulated during the cell cycle (Tsuchiya et al., 1998), implicating that MSG5 might interact with and/or phosphorylate SFH1 in *S. sclerotiorum*, and this interaction relationship might contribute to the normal cell cycle and hyphal growth. We observed a transcriptional reduction of *Sssmg5* in *Sssfh1*-silenced strains (**Supplementary Figure S6**), though it is not proved at protein level, which suggests a potential feedback regulation between MSG5 and SFH1. MSG5 interacts with SLT2, another MAPK, which is involved in the cell wall integrity in yeast (Palacios et al., 2011). Undoubtedly, hyphal cell wall integrity is important for fungi to respond to the abiotic stress (Yu et al., 2012). Meanwhile, osmotic stress or high levels of ROS can cause stress in fungi resulting in growth inhibition (Heller and Tudzynski, 2011; Ryder et al., 2013). We observed decreased sensitivity to both osmotic and oxidative stress in RNAi strains when they were inoculated on the PDA media in the presence of NaCl or H_2_O_2_ (**Figure 6**). Furthermore, we found that, in RNAi strains, less ROS production or accumulation happens when the expression of *Sssfh1* is reduced (**Figure 7**).

Reactive oxygen species biogenesis and scavenging is important for proper ROS accumulation in fungus (Schouten et al., 2002). NADPH oxidases (NOX) produce ROS to regulate different cellular functions, including cell proliferation, cell differentiation and signal transduction (Foreman et al., 2003; Lambeth, 2004; Scott and Eaton, 2008). In another sclerotia-producing fungus, *B. cinerea* possesses two genes encoding catalytic subunits of NOX regulating the ROS production (Segmuller et al., 2008), and the proper accumulation of ROS is vital for full virulence (Siegmund et al., 2015). In human beings, GATA-6 has been implicated in the transcriptional regulation of *NOX1* (Valente et al., 2008), while in *B. cinerea*, BcLTF1 as a GATA TF is required to cope with oxidative stress and to maintain the ROS homoeostasis (Schumacher et al., 2014). On the other hand, fungi have the oxidative stress response (OSR) mechanisms to cope with the elevated intracellular ROS levels, and these mechanisms include enzymatic systems, such as that the glutathione peroxidase (GPX) is responsible for ROS scavenging (Heller and Tudzynski, 2011). In this study, we observed less ROS accumulation in *Sssfh1*-silenced strains compared to the WT strain, and the genes accounting for ROS biosynthesis (*Ssnox1* and *Ssnox2*) were down-regulated, whereas the gene for ROS scavenging, *Ssgpx*, was transcriptionally up-regulated (**Figure 7D**). It is likely that the less accumulation of ROS is because of that we reduced the expression of *Sssfh1* by gene silencing, which manipulates the gene expressions in the context of proper endogenous ROS accumulation. It is well defined that appropriate ROS accumulation in fungus is vital for normal hyphal growth, pathogenicity or even sclerotia producing (Scott and Eaton, 2008; Segal and Wilson, 2018). In *B. cinerea*, BcNOXA and BcNOXD, two major enzymatic producers of ROS, are involved in pathogenicity and formation of sclerotia and conidia (Siegmund et al., 2015). In the root pathogen *Verticillium dahliae*, NoxB is indispensable for pathogenicity and it co-localizes with VdPls1 in hyphopodium, an infection structure, to mediate ROS production to regulate Ca^2+^-dependent polarized penetration peg formation (Zhao et al., 2016). We assume that the fungi can grow normally in the presence of certain amount of ROS and the growth would be affected if the ROS homeostasis is interrupted (Segal and Wilson, 2018). Similarly, the fungi could endure certain levels of ROS accumulation from both endogenous biogenesis and exogenous treatment. In this study, there is less ROS accumulation in RNAi strains, which in turn can tolerate the exogenous ROS produced by osmotic or oxidative reagents (**Figures 6, 7**). One possibility is that more ROS is required in the silenced fungal strains, which are less ROS-accumulated, to reach a certain level of detrimental ROS effect, as revealed by increased tolerance to exogenous ROS caused by osmotic and oxidative stresses (**Figure 6**). On other hand, the increased expression of *Ssgpx* in RNAi strains could help to scavenge the excess ROS generated by osmotic and oxidative stresses, which cause ROS accumulation or ROS diffusion directly into the fungal hyphal cells.

The successful host infection by *S. sclerotiorum* is highly related to infection cushion formation and function (Huang et al., 2008), which is associated with proper ROS accumulation in hyphal cells (Marschall and Tudzynski, 2016). In our previous study, deletion of *Ssnsd1*, another GATA-box containing TF in *S. sclerotiorum*, results in mutants that fail to produce compound appressoria (infection cushion) and exhibit penetration failure and virulence defects (Li et al., 2017). We observed slower hyphal growth, less infection cushion formations, less ROS accumulations, and reduced virulence on host plants, in the *Sssfh1*-silenced strains, which is in agreement of the importance of normal infection cushion formation and proper ROS accumulation in the process of host infection (Egan et al., 2007). In *S. sclerotiorum*, silencing *Ssnox1* expression results in reduced ROS levels and attenuated virulence (Kim et al., 2011), meanwhile, disruption of *BcNOP53*, encoding a pre-rRNA processing factor, significantly reduced the ROS production in *B. cinerea* hyphae leading to failure in cushion-like infection structure formation, eventually failure in host penetration (Cao et al., 2018). It is likely that less ROS accumulation causes less production of infection cushion, leading to low efficiency penetration and virulence defects. However, the leaves that were wounded prior to infection still showed no significant difference in infection efficiency compared with the unwounded leaves when inoculated with *Sssfh1*-silenced strains, suggesting that penetration deficiency caused by less infection cushion formation is not the only factor accounting for the loss of pathogenicity. In *S. sclerotiorum*, loss of pathogenicity might be caused by a combination of proper growth, development or accumulation of hyphae, infection cushion, or ROS rather than a single factor. As the regulatory connections between ROS, hyphal growth, infection cushion, and pathogenicity are not well understood in *S. sclerotiorum*, unique regulators and links may be elucidated as we gain more information on the suite of regulators affecting these developmental stages, such as *Sssfh1* and their downstream regulated genes.

## Materials and Methods

### Fungal Strains, Culture Conditions, and Plant Materials

Wild-type *S. sclerotiorum* isolate 1980 (Godoy et al., 1990; Derbyshire et al., 2017) and RNA-silenced strains were cultured, unless otherwise stated, on PDA medium at 25°C. PDA medium supplemented with 100 ug/mL geneticin (Roche, China) was utilized to culture the transformants. In the abiotic stress assay, PDA medium was supplemented with 1 M NaCl or various concentrations of H_2_O_2_ (5, 15, and 25 mM). Hyphae stocks were maintained as desiccated mycelia-colonized filter paper at -20°C and as dry sclerotia at 4°C (Wang et al., 2016). Soybean, common bean and tomato plants were grown in the glasshouse under natural sunlight, with temperature in the 22–25°C range.

### Bioinformatics Analysis of SsSFH1

In our previous study, we found based on PFAM analysis that there are nine GATA-box-containing proteins encoded in the *S. sclerotiorum* genome, in which a gene (SS1G_01151) encoding a TF with a GATA-box and an SNF5 domain was isolated in this study. To analyze the relationships among the documented GATA-box and SNF5 domain-containing proteins in different species, phylogenetic tree was constructed using MEGA software (version 7.05) with the maximum likelihood method using 1000 bootstrap replicates to ascertain the reliability of a given branch pattern in the NJ tree. Meanwhile, detailed protein sequence alignment using *S. sclerotiorum*, SsSFH1 (XP_001596958.1), *F. fujikuroi*, FfSFH1 (KLP21684.1), *P. subalpina*, PsSFH1 (CZR60918.1), *R. agropyri*, RaSFH1 (CZT00122.1), and *S. pombe*, SpSFH1 (NP_588001.1) was performed using BioEdit program.

### Generation of RNAi Constructs

The vector pSilent-Dual1 (Nakayashiki et al., 2005; Nguyen et al., 2008; Fan et al., 2017) was used as backbone to generate *S. sclerotiorum* RNAi constructs. In detail, Target 1 (527 bp) and Target 2 (358 bp) of *Sssfh1* coding fragments were amplified with two pairs of specific primers (The primers are listed in **Supplementary Table S1**) from the synthesized *S. sclerotiorum* cDNA and were then ligated into pSilent-Dual1. The resulting constructs were confirmed by sequencing.

### Transformation and Evaluation of RNAi Strains

Protoplasts of *S. sclerotiorum* were prepared and the quality of protoplasts was examined by microscopy as described previously (Qu et al., 2014). The RNAi constructs were introduced into the prepared protoplasts by a PEG-mediated transformation method (Rollins, 2003). Colonies regenerated through the selective medium were transferred to PDA with 100 ug/mL geneticin. Transformants were cultured and purified at least three times on PDA containing the corresponding antibiotics using freshly grown hyphal tips. The transformants were verified by PCR with specific primers (Gene-F/Gene-R) using genomic DNA as template (Fan et al., 2017). Three individual transformants from each vector were used for phenotypic assay. The silencing efficiency in each RNAi strain was examined by qRT-PCR.

### Hyphal and Sclerotial Morphology and Hyphal Growth Rate Observation

To investigate the influence of silencing *Sssfh1* on hyphal growth and morphology, WT and RNAi strains were cultured on PDA medium at 25°C. Colony diameters of each strain, which was inoculated in the center of a 9 cm petri dish with an agar mycelium plug (5 mm in diameter) derived from the growing margin of the PDA culture, were measured over time to determine the radial mycelial growth on PDA plates. Hyphal morphology was observed by light microscopy (Nikon Eclipse 80i digital microscopy, Melville, NY, United States). Two weeks after inoculation, sclerotia were collected and the numbers were counted.

### Pathogenicity Assay

To evaluate the impact of RNA silencing on pathogenicity, 7-week-old soybean leaves and 5-week-old common bean leaves were detached and placed in 15 cm petri dishes with moist filter paper. Those leaves were inoculated with a 5 mm PDA-colonized agar plug of either WT or *Sssfh1*-silenced strains, and were placed in a high-humidity chamber at 25°C for 3 days. The diameters of the necrotic lesions were measured and the pathogenicity assays were performed three times. The photographs were taken with a Canon EOS 550D (Canon, Tokyo, Japan) camera in this study.

### Protein–Protein Interaction Assays

The Y2H assay was performed using a GAL4-based Y2H system-Matchmaker^TM^ Two-Hybrid System 3 (CLONTECH, Palo Alto, CA, United States). The *Sssfh1* was amplified and ligated into pGBKT7 (*Sal* I and *Pst* I). Previously, Li and Rollins redefined six distinct and sequential stages of sclerotial development and seven distinct and sequential stages of apothecia development (Li and Rollins, 2009). Based on this definition, total RNA isolated from six sequential stages of sclerotial and seven sequential stages of apothecia and hypha were pooled, and the full-length cDNA library was prepared by Invitrogen Corporation (Shanghai, China). The full-length cDNAs encoding candidate proteins were cloned into pGADT7 (primers are listed in **Supplementary Table S1**). To test the specificity of the interaction, the pGBKT7-based constructs and the pGADT7-based constructs were co-transformed into yeast strain AH109. The transformants were assayed for growth on SD (synthetic dropout)/-Trp-Leu-His-Ade plates with X-α-Gal for β-galactosidase test (Yu et al., 2012).

For BiFC assay, *Sssfh1, Ssmsg5*, and *Ssfus3/Sskss1* were put in frame of separated halves of YFP (Walter et al., 2004; Wei et al., 2016), resulting in pSAT4-*Sssfh1*-nYFP, pSAT4-*Sssmg5*-cYFP, and pSAT4-*Ssfus3/Sskss1*-cYFP (Tzfira et al., 2005). These constructs were subsequently co-transformed into *Arabidopsis* protoplasts (Li et al., 2010). After 12–16 h of incubation, YFP fluorescence signal was observed under a FM-100 inverted fluorescence microscope (Carl Zeiss, German).

### ROS Detection

To detect H_2_O_2_ accumulation, different strains were grown on solid complete medium (CM) or on solid CM covered with cellophane for 48 h. 0.5 cm PDA-colonized agar plugs (*n* = 6), fresh mycelia (20 mg) of either WT or *Sssfh1*-silenced strains were obtained and placed in 24-well plate and were flooded with 1 ml DAB solution (0.5 mg/ml DAB in 100 mM of citric acid buffer, pH 3.7) (Schumacher et al., 2014). Samples were incubated for 2 h in darkness at 25°C. The colorless DAB is oxidized by H_2_O_2_ in the presence of fungal peroxidases resulting in a brown coloration of the staining solution (Schumacher et al., 2014). To detect O_2_^-^ accumulation, different strains grown on solid CM covered with cellophane for 36 h were incubated for 20 min in NBT staining solution (0.5 mg/ml nitrotetrazolium blue chloride). Samples were analyzed by using Nikon Eclipse 80i fluorescence microscope system. The colorless substrate NBT is converted to a blue precipitate in the presence of O_2_^-^ (Schumacher et al., 2014).

### Gene Expression Analysis by qRT-PCR

The gene expression profiles in *S. sclerotiorum* were analyzed by qRT-PCR. The transformants and WT strain were cultured on PDA overlaid with cellophane for 2 days. Total RNA was isolated using TRIzol reagent according to the manufacturer’s instructions (Invitrogen, Carlsbad, CA, United States). One microgram of total RNA was subjected to cDNA synthesis using PrimeScript^TM^ RT Reagent Kit with gDNA Eraser (TaKaRa, Dalian, China). qRT-PCR was performed using the primer pairs listed in **Supplementary Table S1**. To assure the accurate use of appropriate reference gene, the expression levels of actin, tubulin, and histone (**Supplementary Table S1**) in *S. sclerotiorum* were evaluated in each qRT-PCR assay and their mean value was used as internal reference (Gutierrez et al., 2008). PCRs were conducted as follows: 30 s at 95°C, then 40 cycles each consisting of 5 s at 95°C, 40 s at 60°C and 15 s at 95°C, followed by 1 min at 60°C and 15 s at 95°C. The relative expression levels were calculated by 2^-ΔΔCT^ method (Schmittgen et al., 2000). Each RT-PCR experiment was performed at least three times.

## Author Contributions

LL, GY, and HP planned and designed the research and wrote the manuscript. All authors performed the experiments and analyzed the data.

## Conflict of Interest Statement

The authors declare that the research was conducted in the absence of any commercial or financial relationships that could be construed as a potential conflict of interest. The reviewer JM-Á and handling Editor declared their shared affiliation.
