# Supplementary material for: Sssfh1, a Gene Encoding a Putative Component of the RSC Chromatin Remodeling Complex, Is Involved in Hyphal Growth, Reactive Oxygen Species Accumulation, and Pathogenicity in Sclerotinia sclerotiorum
Source: Front Microbiol. 2018 Aug 7;9:1828. doi: 10.3389/fmicb.2018.01828 (PMC6090059; doi:10.3389/fmicb.2018.01828)
Supplement: Supplementary file 1 [file Presentation_1.pdf]

Table S1. Primers used in this study.

	Primers	Sequence (5'to3')
RNAi Primers (*Hind*III)	*Sssfh1*-T1-F	CCCAAGCTTGGAGATCAGCAAGCAGAT
	Sssfh1-T1-R	CCCAAGCTTAACCACCACTTTCACATG
	Sssfh1-T2-F	CCCAAGCTTTCATACAACCAGCGACAG
	Sssfh1-T2-R	CCCAAGCTTGGCGTGAAGGAAGGAATA
Y2H Primers	*Sssfh1*-BD-F-*Sal* I	GCGTCGACCTATGACTACTCGCAACCC
	Sssfh1-BD-R-*Pst* I	AACTGCAGTCATCTGTAATCACTTGAGC
	Ssmsg5-AD-F-*Sac* I	GATACGGGATCCATCGAGCTATGCCATCAGCAGCT
	Ssmsg5-AD-R-*Sac* I	ATTCATCTGCAGCTCGAGCTTCAAAGGAAGTCATCGATAG
BiFC Primers	*Sssfh1*-F-YFP-N	GTCGACGGTACCGCGGGCCCATGACTACTCGCAACCCT
	Sssfh1-R-YFP-N	TTGCTCACCATCAGGATCCCTCTGTAATCACTTGAGCGGA
	Ssmsg5-F-YFP-C	GTCGACGGTACCGCGGGCCCATGCCATCAGCAGCT
	Ssmsg5-R-YFP-C	TGCACGCTGCCCAGGATCCCAAGGAAGTCATCGATAGATC
qRT-PCR Primers	qRT*Ssnox1*-F	CGCAATCCAGATTCACTATAC
	qRT*Ssnox1*-R	GACGAAGCATCCAGTAGC
	qRT*Ssnox2*-F	CATTCACCGTCACTTATGC
	qRT*Ssnox2*-R	TACAATGCCGTTCAGAGG
	qRT*Ssmsg5*-F	TCCTCCTTCCAAACTGCC
	qRT*Ssmsg5*-R	ACCCTTTTCCCCTCCTTAG
	qRT*Ssgpx*-F	GGTCTTCTCGGTCTTCAAC
	qRT*Ssgpx*-R	TCTCATTCAAAGCCTCCAAA
qRT-PCR Housekeeping genes Primers	Actin-F	GAATGTGTAAGGCCGGTTTCGC
	Actin-R	CATCCCAGTTGGTGACGACACC
	Histone-F	GGCTCGTACCAAGCAAACTG
	Histone-R	GAAGTCTTGGGCGATTTCAC
	Tubulin-F	GGTGAGCATGGTCTTGACGG
	Tubulin-R	CCCTCAGCCTCACGACGAAC
Geneticin gene Primers	Gene-F	TGTCCGGTGCCCTGAATGAACT
	Gene-R	GCCGCCAAGCTCTTCAGCAATAT

Figure S1. Sequence analysis of GATA-box domain containing proteins in *S. sclerotiorum*
A: Phylogenetic analysis of nine GATA-box domain-containing proteins in *S. sclerotiorum*. Phylogenies were speculated using MEGA (version 7.05) program with the Neighbor-Joining method (1000 bootstrap replicates) to create an unrooted phylogenetic tree. The GATA-boxes were highlighted in red, the PAS-domains were shaded in yellow and the SNF5 domain was in green.
B: BLASTN search was used to show low nucleotide sequence similarities between *Sssfh1* and others genes in *S. sclerotiorum* genome.
Figure S2. Identification of selective marker insertions by PCR
RNAi mutants were inoculated on PDA media for two days and the hyphae were collected for DNA preparation. Conventional PCR were performed using primers (Gene-F/Gene-R) specific for Geneticin resistance genes and produced 650 bp PCR products from RNAi mutants. M: DNA molecular size marker; W: wild type strain; lanes 1–3: putative transformants *Sssfh1*-T1-25, *Sssfh1*-T1-27 and *Sssfh1*-T1-45; lanes 4–6: putative transformants *Sssfh1*-T2-13, *Sssfh1*-T2-1 and *Sssfh1*-T2-25.
Figure S3. Effect of *Sssfh1* silencing on sclerotium development in culture.
A: Comparison of sclerotia morphology and size among the WT and the *Sssfh1*-silenced transformants. The scale bar is 0.5 cm.
B: Average sclerotium numbers collected from 5 cm culture plates of the WT and the *Sssfh1*-silenced transformants.
C: Average sclerotium dry weight collected from 5 cm culture plates of the WT and the *Sssfh1*-silenced transformants.
In all experiments, at least three independent replications were performed. WT and selected RNAi mutant strains were cultured on PDA medium at 25℃. Two weeks after inoculation, sclerotia were collected and counted. Each data point represents the means ± SD from three biological repeats (n = 3).
Figure S4. *Sssfh1* is required for full virulence of *S. sclerotiorum*.
A: Fungal virulence was affected in the RNAi strains on tomato leaves. Symptoms were photographically documented at 36 hpi. The necrosis on tomato leaves caused by different strains were shown from adaxial (upper panel) and abaxial (lower panel).
B: Fungal virulence were affected in the RNAi strains on wounded common bean leaves. Mycelial plugs of the indicated strains were inoculated on wounded common bean leaves and the disease symptoms were observed and recorded by photograph at both 24 and 48 hpi.
Figure S5. Observation of the lesion characteristics in the *Sssfh1*-silenced transformants and the WT strain.
A: The detached leaves were inoculated with mycelial plugs from different strains (diameter=5 mm) and pictures were taken at 24 hpi.
B: The infected leaves (A) plant tissues were decolorized and the sites of infection lesions were observed under microscopy system. Scale bar=10 um.
C: The enlarged infection tissues are presented. Subcuticular vesicles and inter- and intracellular infectious hyphae are highlight by red arrow, infection area with *Sssfh1*-

silenced transformants are highlighted by dashed lines circle. Others dark spots are trichomes.

Figure S6. Expression of *Ssmsg5* in different RNAi strains.

The expression of *Ssmsg5* gene was investigated in hyphae tissue in RNAi and WT strains. Each data point represents the means ± SD from three biological repeats ($n = 3$) (one-way ANOVA, ** indicates significance at $p < 0.01$).

Figure S7. Relative inhibition rate of hyphae growth in the PDA with NaCl.

Both WT and RNAi strains were inoculated on PDA media that was supplemented with osmotic reagents, 1M NaCl was used to mimick osmotic stress environment. The colony diameters of different strains were counted after incubation at 25℃ for 3 days. Each data point represents the means ± SD from three biological repeats ($n = 3$)

A

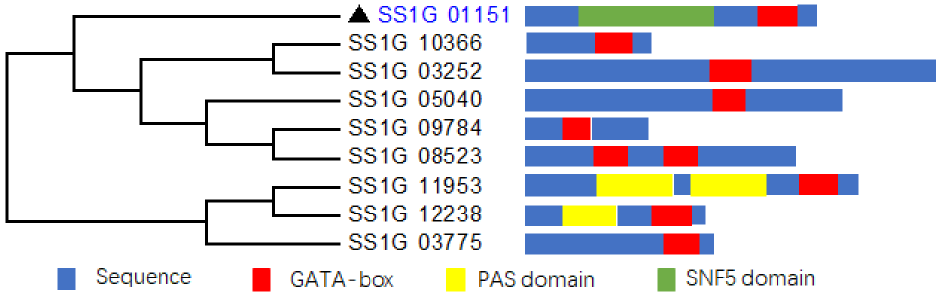

B

Gene ID	Transcript ID	Score	E-Value
SS1G_01151	SS1G_01151-t26_1	3236	0E0
SS1G_03063	SS1G_03063-t26_1	39.2	0.045E0
SS1G_07790	SS1G_07790-t26_1	37.4	0.16E0
SS1G_02576	SS1G_02576-t26_1	35.6	0.54E0
SS1G_04913	SS1G_04913-t26_1	35.6	0.54E0
SS1G_12236	SS1G_12236-t26_1	35.6	0.54E0
SS1G_13725	SS1G_13725-t26_1	35.6	0.54E0
SS1G_13802	SS1G_13802-t26_1	35.6	0.54E0
SS1G_03591	SS1G_03591-t26_1	33.7	1.9E0

Figure. S1

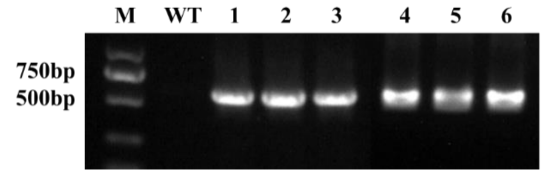

Figure. S2

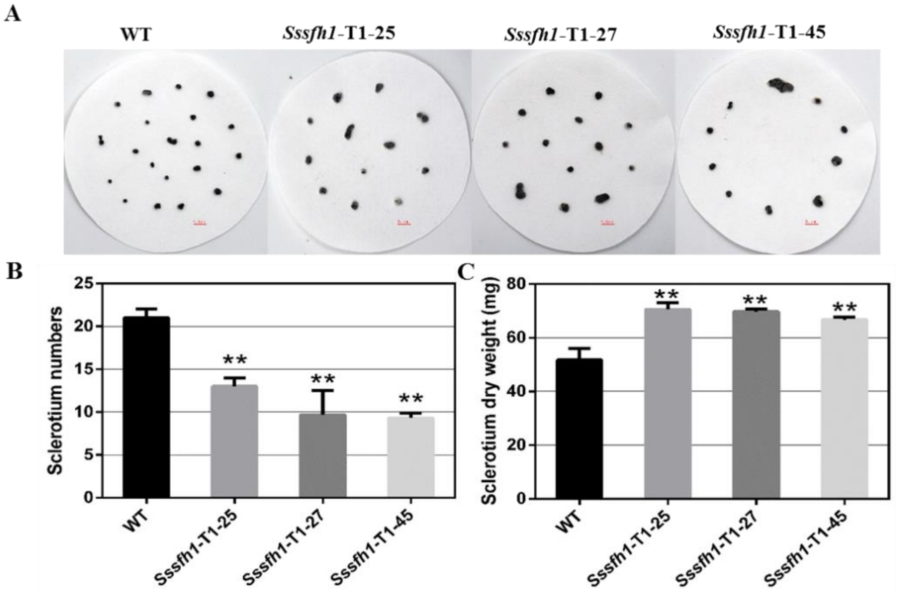

Figure. S3

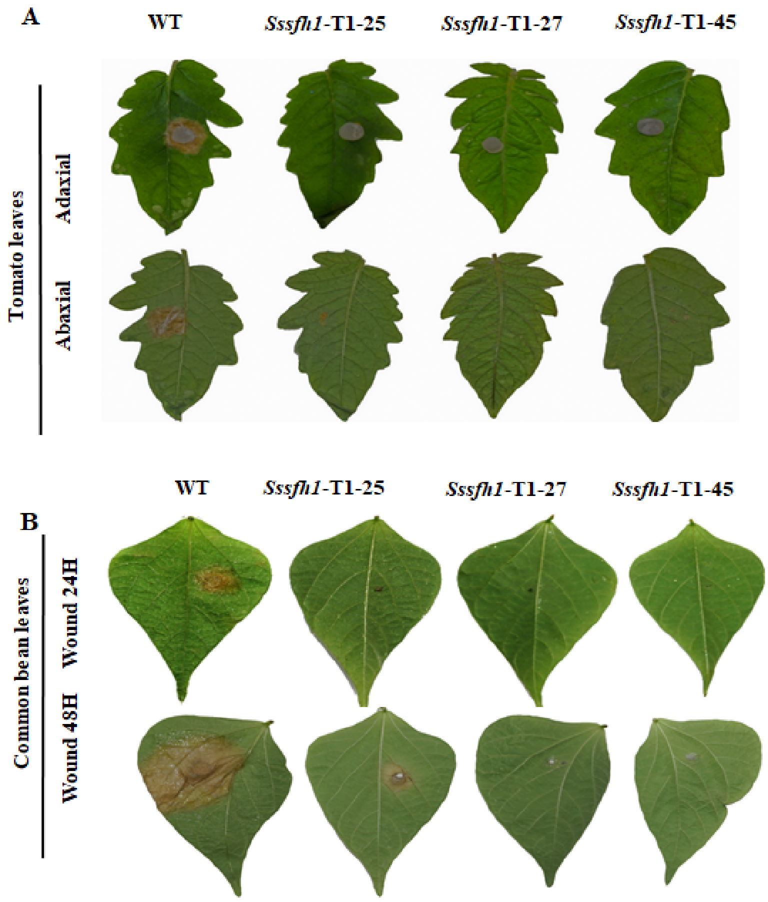

Figure. S4

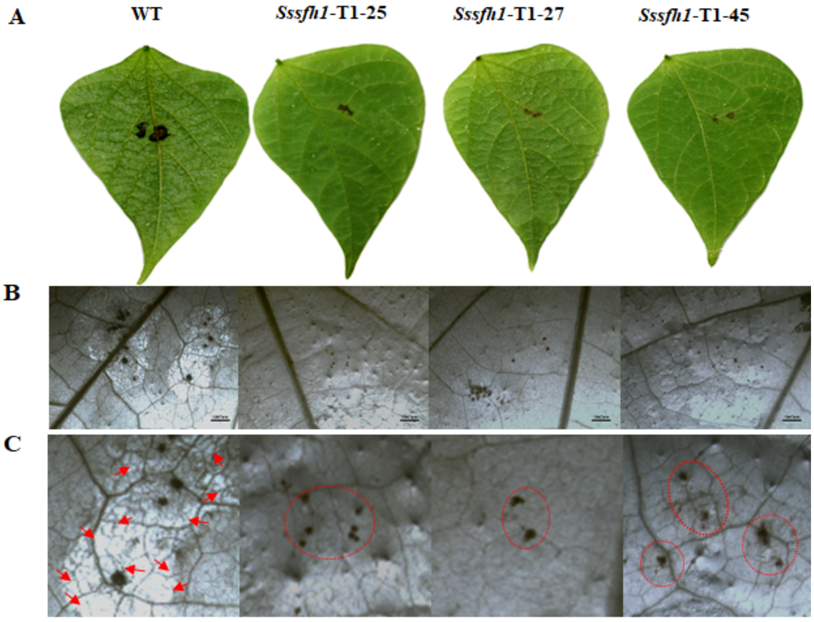

Figure. S5

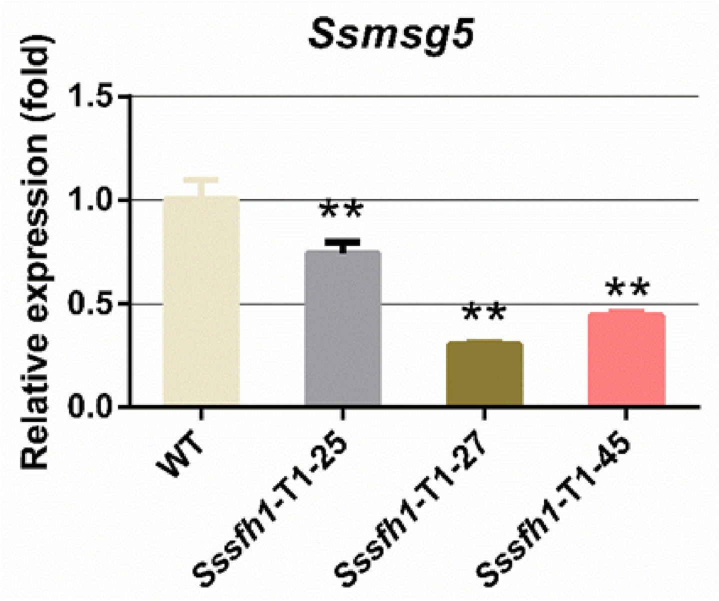

Figure. S6

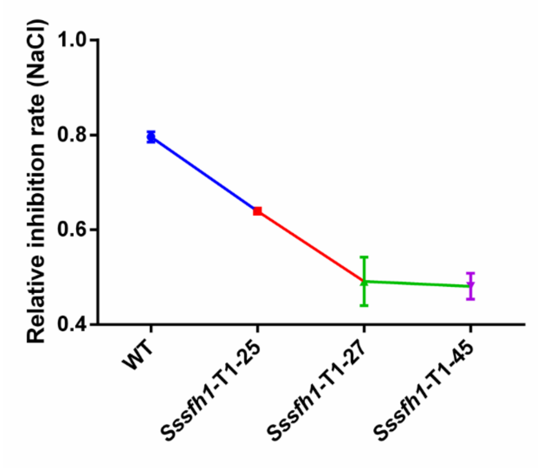

Figure. S7
